# Unveiling the molecular mechanisms: dietary phytosterols as guardians against cardiovascular diseases

**DOI:** 10.1007/s13659-024-00451-1

**Published:** 2024-05-09

**Authors:** Nasreddine El Omari, Saad Bakrim, Asaad Khalid, Ashraf N. Abdalla, Mohamed A. M. Iesa, Kawtar El Kadri, Siah Ying Tang, Bey Hing Goh, Abdelhakim Bouyahya

**Affiliations:** 1 High Institute of Nursing Professions and Health Techniques of Tetouan, Tetouan, Morocco; 2 Geo-Bio-Environment Engineering and Innovation Laboratory, Molecular Engineering, Biotechnology and Innovation Team, Polydisciplinary Faculty of Taroudant, Ibn Zohr University, 80000 Agadir, Morocco; 3 Substance Abuse and Toxicology Research Center, Jazan University, P.O. Box: 114, 45142 Jazan, Saudi Arabia; 4 Medicinal and Aromatic Plants and Traditional Medicine Research Institute, National Center for Research, P. O. Box 2404, Khartoum, Sudan; 5 Department of Pharmacology and Toxicology, College of Pharmacy, Umm Al-Qura University, 21955 Makkah, Saudi Arabia; 6 Laboratory of Human Pathologies Biology, Faculty of Sciences, Mohammed V University in Rabat, 10106 Rabat, Morocco; 7 Department of Chemical Engineering, School of Engineering, Monash University Malaysia, Jalan Lagoon Selatan, 47500 Bandar Sunway, Selangor Darul Ehsan Malaysia; 8 Biofunctional Molecule Exploratory Research Group, School of Pharmacy, Monash University Malaysia, 47500 Bandar Sunway, Malaysia; 9 Sunway Biofunctional Molecules Discovery Centre (SBMDC), School of Medical and Life Sciences, Sunway University, 47500 Sunway City, Malaysia; 10 Faculty of Health, Australian Research Centre in Complementary and Integrative Medicine, University of Technology Sydney, Ultimo, NSW 2007 Australia; 11 Department of Physiology, Al Qunfudah Medical College, Umm Al Qura University, Mecca, Saudi Arabia

**Keywords:** Phytosterols, *β*-sitosterol, Stigmasterol, Cardiovascular disease, Nutritional protection

## Abstract

Until recently, the main pharmaceuticals used to control cholesterol and prevent cardiovascular disease (CVD) were statin-related drugs, known for their historical side effects. Therefore, there is growing interest in exploring alternatives, such as nutritional and dietary components, that could play a central role in CVD prevention. This review aims to provide a comprehensive understanding of how natural phytosterols found in various diets combat CVDs. We begin with a description of the overall approach, then we explore in detail the different direct and indirect mechanisms that contribute to reducing cardiovascular incidents. Phytosterols, including stigmasterol, *β*-sitosterol, ergosterol, and fucosterol, emerge as promising molecules within nutritional systems for protection against CVDs due to their beneficial effects at different levels through direct or indirect cellular, subcellular, and molecular mechanisms. Specifically, the mentioned phytosterols exhibit the ability to diminish the generation of various radicals, including hydroperoxides and hydrogen peroxide. They also promote the activation of antioxidant enzymes such as superoxide dismutase, catalase, and glutathione, while inhibiting lipid peroxidation through the activation of Nrf2 and Nrf2/heme oxygenase-1 (HO-1) signaling pathways. Additionally, they demonstrate a significant inhibitory capacity in the generation of pro-inflammatory cytokines, thus playing a crucial role in regulating the inflammatory/immune response by inhibiting the expression of proteins involved in cellular signaling pathways such as JAK3/STAT3 and NF-κB. Moreover, phytosterols play a key role in reducing cholesterol absorption and improving the lipid profile. These compounds can be used as dietary supplements or included in specific diets to aid control cholesterol levels, particularly in individuals suffering from hypercholesterolemia.

**Graphical Abstract:**

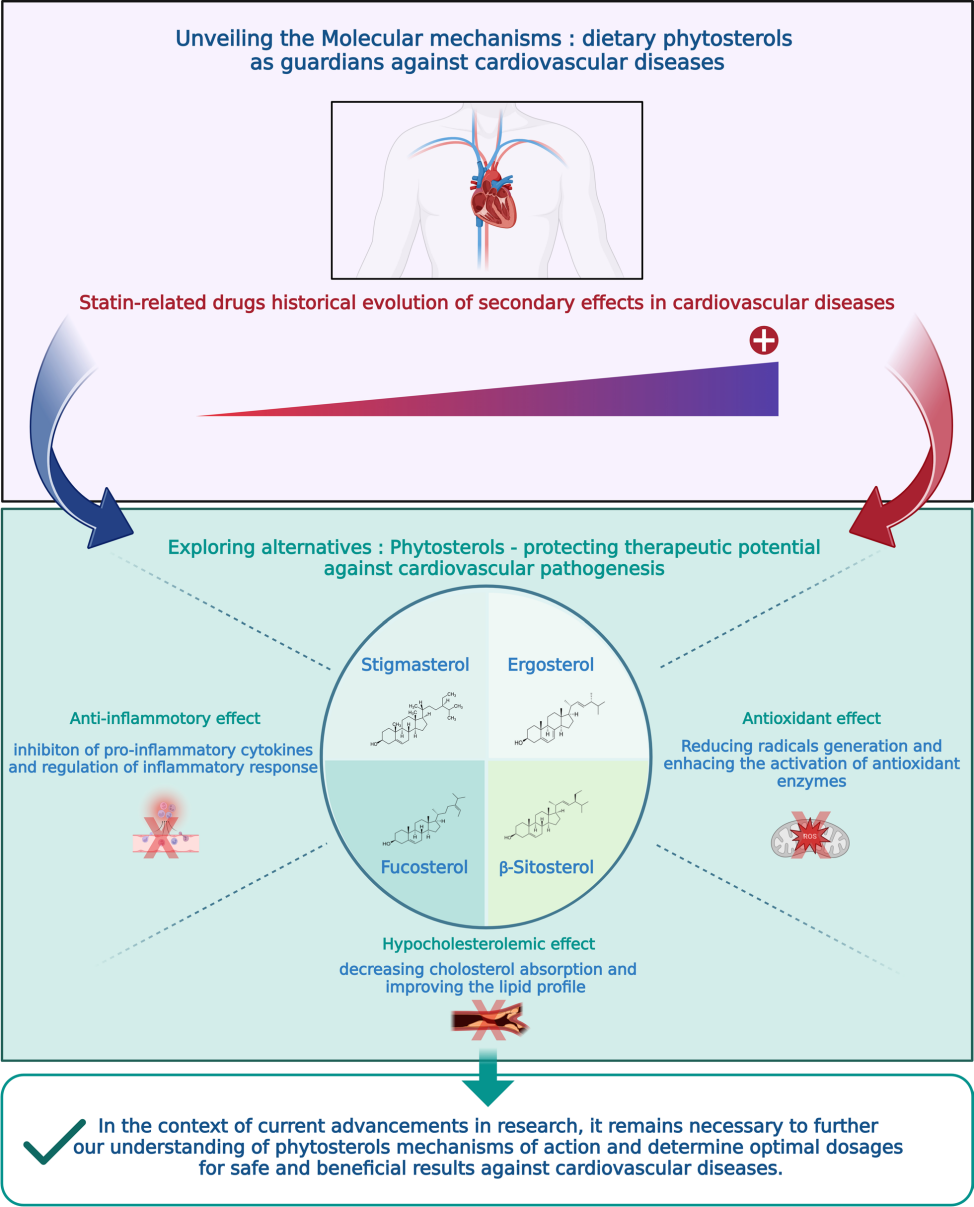

## Introduction

Cardiovascular diseases (CVDs) remain among the most prevalent contributors to global mortality [1–3]. In 2019, an estimated 17.9 million people died from these diseases, accounting for 32% of all deaths worldwide, and 85% of these deaths were related to stroke or myocardial infarction [4]. Several risk factors contribute to the occurrence of CVDs, including chronic inflammation, hyperlipidemia, hypertension, obesity, sedentary, overweight, diabetes mellitus, and genetic predisposition [5–7]. Regardless of significant attempts to address these traditional risk factors, other potential cardiovascular risks are emerging. Oxidative stress specifically refers to the process of chemical imbalance between reacting substances, including reactive oxygen species (ROS) and antioxidants. High levels of reactive species destroy lipoproteins and lipids, and alterations of these components induced by oxidative stress have been linked to the onset and progression of atherosclerotic CVDs. The hypothesis is that this phenomenon is mainly due to the oxidation of low-density lipoproteins (ox-LDL), with other components (proteins, DNA, and lipids) also being considered [8, 9]. On the other hand, for several decades, most of the treatments available for the fundamental prevention of CVDs and for lowering cholesterol levels are drugs in the statin class, acting by inhibiting HMG-CoA reductase (3-hydroxy-3-methyl-glutaryl-CoA reductase), a vital enzyme in the production of cholesterol. Although their effectiveness has been demonstrated, statins are linked to adverse events, the most frequent of which are cramps, myalgia and myopathy or neuromuscular junction disorders, and occasionally, peripheral neuropathies [10]. Furthermore, due to of their numerous comorbidities and use of additional drugs that could adversely interfere with statins, elderly individuals are more susceptible than younger ones to develop statin adverse reactions [11].

In this context, researchers have been criticized for giving excessive attention to academic research on alternatives, specifically regarding the potential role of plant-based diets in the early prevention of CVDs. Indeed, there is a significant link between diet and increased risk of these types of diseases. Diets high in cholesterol and saturated fat are major contributors to atherosclerosis and CVDs, leading to the emergence of the so-called “diet-heart hypothesis” [7]. For this reason, many diets have been developed to reduce the risk of CVDs. Vegetarian diets, such as the Mediterranean diet, have been consistently reported to decrease plasma cholesterol levels, blood pressure, and fasting blood glucose [12, 13]. These types of diets are rich in phytosterols which represent plant-derived compounds that are structurally similar to cholesterol. They are found primarily in unprocessed vegetable oils, grains, olive oil, and nuts. Each day, the average Western diet includes about 400 mg of cholesterol and about 400 mg of plant sterols. Phytosterols, unlike cholesterol, are not produced by humans and their only source is food [4]. In fact, the most prominent and significant attribute of phytosterols is their potential to effectively reduce the level of cholesterol in the blood, which in turn may play a role in minimizing the risk of CVD. The mechanisms that explain this property have been largely investigated and focus on pathways directly involving cholesterol, such as gene regulation, protein-mediated absorption, interaction with digestive enzymes, and intestinal solubility. Phytosterols such as *β*-sitosterol, *β*-sitostanol, stigmasterol, and ergosterol were found to reduce the levels of several markers of atherosclerosis risk [14–17]. Furthermore, these bioactive molecules also exert other mechanistic pathways related to inflammation and oxidative stress. In this sense, multiple investigations on cellular antioxidant processes have demonstrated that phytosterols are capable of stimulating antioxidant enzymes to decrease the generation of ROS and avoid damage caused by oxidative stress. In fact, stigmasterol, *β*-sitosterol, ergosterol, and fucosterol all promote the stimulation of some antioxidant enzymes, including superoxide dismutase (SOD), catalase (CAT), glutathione (GSH), and others [18–21]. Numerous studies have examined the mechanisms of action underlying the anti-inflammatory potential of phytosterols. Indeed, a large majority of these investigations have demonstrated that these natural substances act at various levels to reduce inflammation through cellular, subcellular, and molecular pathways. In one instance, the inducible nitric oxide synthase (iNOS) was inhibited by the presence of *β*-sitosterol, stigmasterol, ergosterol, and fucosterol [22, 23]. Additionally, as an anti-inflammatory approach, numerous authors have focused on the suppression of cytokine activity or their mRNA levels. All of the phytosterols described above demonstrated considerable inhibitory capacity in the generation of pro-inflammatory cytokines, whether in vivo or in vitro [24–26]. Enhancement of immunological and endothelial activities, modification of certain signaling pathways involved in the control of inflammation and lipid metabolism, and other effects of phytosterols have also been directly linked to protection from these disorders [27].

Although several studies have reported the use of phytosterols in the prevention of CVDs, the precise mechanisms by which these compounds act to reduce endogenous cholesterol are not yet well elucidated and defined. In this context, our review first proposes to highlight the action of phytosterols against CVDs from a holistic perspective, and then to outline the different direct and indirect mechanisms linked to the improvement of cardiovascular attacks, in particular a comprehensive and detailed analysis of the antioxidant, anti-inflammatory, hypolipidemic and immunomodulatory properties of phytosterols.

## Protective roles of phytosterols against cardiovascular diseases

**Fig. 1:**
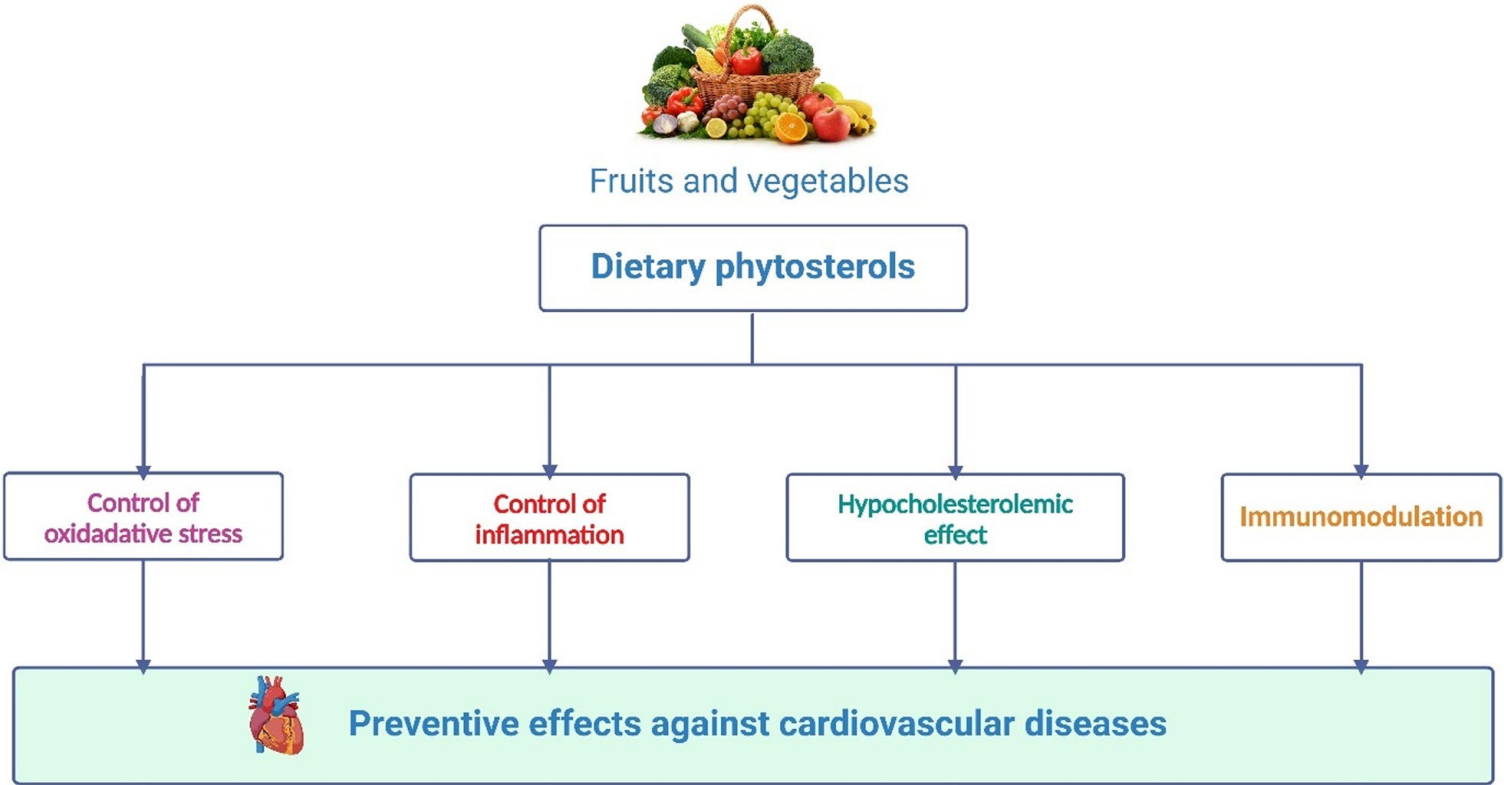
Schematic representation of mechanistic pathways involved in the protection against cardiovascular diseases by phytosterols. CAT: catalase; SOD: superoxide dismutase; GSH: glutathione; GPx: glutathione peroxidase; GR: glutathione reductase; APX: ascorbate peroxidase; PPO: polyphenol oxidase; POD: peroxidase; NO: nitric oxide; Cyt c: Cytochrome C; LP: lipid peroxidation; TBARS: thiobarbituric acid reactive substances; HPO: hydroperoxides; LOX: lipoxygenase; Nrf2: nuclear factor-erythroid 2-related factor 2; MCP-1: monocyte Chemoattractant Protein-1; ROS: reactive oxygen species; CVD: cardiovascular diseases; LDL-C: low-density lipoprotein cholesterol; AKT: protein kinase B; JAK 3: janus kinase 3; STAT3: signal transducer and activator of transcription 3; ICAM-1: intracellular adhesion molecule-1; VCAM-1: vascular cell adhesion molecule-1; NF-κB: nuclear factor-κB; IL: Interleukin; TNF-α: tumor necrosis factor-α; ACAT-1: acyl-CoA: cholesterol acyltransferase-1; VEGF: vascular endothelial growth factor; MAPK: mitogen-activated protein kinase; ERK: extracellular signal-regulated kinase; TLR 4: toll-like receptor 4; ACC: acetyl-CoA carboxylase; FAS: FA synthase; FAT: FA translocase; PPARγ: peroxisome proliferator-activated receptor γ; iNOS: inducible nitric oxide; PGE 2: prostaglandin E 2; COX-2: cyclooxygenase-2

Phytosterols are plant-derived compounds that are structurally similar to cholesterol and are known to have a cholesterol-lowering effect in humans. Phytosterols, which are available as supplements or functional foods, are recognized by the European Union as foods, can be purchased without a prescription, and are frequently taken without the guidance of a healthcare provider. Several studies have also suggested that phytosterols may have protective roles against CVDs, which are a leading cause of mortality worldwide. The following are some of the mechanisms through which phytosterols may exert their protective effects (Fig. 1).

### Antioxidant mechanisms

As demonstrated above, it is possible to state that certain phytosterols have a double anti-inflammatory and antioxidant effect, and therefore, additional antioxidant properties. Specifically, several phytosterols have shown promising antioxidant effects via various mechanisms of action classified according to the cellular, sub-cellular, and molecular levels at which they act (Table 1).

**Table 1:** Antioxidant mechanisms of phytosterols

Molecules	Origins	Experimental methods	Key findings	Refs.
*β*-sitosterol	Purchased	PMA-stimulated RAW264.7 macrophages Nitroblue tetrazolium (NBT) method Glutathione (GSH) assay Western blot analysis	Increased GSH/total GSH ratio Reduced H_2_O_2_ and O_2_^•−^ levels Reduced the activity of antioxidant enzymes: catalase (CAT), glutathione peroxidase (GPX), and Mn SOD	[41]
–	*Solanum surattense*	Doses: 10, 15, and 20 mg/kg, p.o Experimental model for diabetes-induced oxidative damage	Decreased NO levels Increased pancreatic antioxidant levels Decreased thiobarbituric acid-reactive substances (TBARS) levels	[33]
–	Purchased	Glucose oxidase (GOX)-induced oxidative stress and lipid peroxidation HT22 hippocampal neuronal cell line DCFH-DA method TBARS assay	Prevented GOX-induced oxidative stress and lipid peroxidation via estrogen receptor (ER)-mediated PI3K/GSK3*β* signaling	[48]
–	Purchased	H9c2 cells and rat hearts Mitochondrial respiration Mitochondrial ROS production Cellular GSH levels Myocardial I/R injury	Stimulated the mitochondrial ATP generation capacity in H9c2 cells Up-regulated cellular GSH redox cycling Protected against hypoxia/reoxygenation-induced apoptosis in H9c2 cells Up-regulated mitochondrial GSH redox cycling in female rat hearts	[44]
–	Purchased	Doses: 10, 20, and 40 mg/kg for 21 days Isoproterenol-induced myocardial infarction in male Wistar rats	Reduced lipid peroxidative product levels Improved the antioxidant status Prevented lipid peroxidation alterations	[45]
–	Purchased	Doses: 20 and 40 mg/kg p.o. for 35 days Ethanol-induced hepatotoxic albino Wistar rats	Decreased the levels of hydroperoxides and TBARS in tissue and plasma Increased GPx, CAT, and superoxide dismutase (SOD) activity Increased the levels of GSH, ceruloplasmin, vitamin C and E	[47]
–	*Eulophia herbacea* and* Eulophia ochreata*	DPPH assay Reducing power assay	Induced antioxidant property through both in vitro methods	[58]
–	Purchased	Doses: 40, 60, 80, and 100 mg/kg p.o. for 42 days ABTS and DPPH radical scavenging assays Hydroxyl radical (OH^−^) and superoxide radical (O_2_^−^) scavenging activities Measurement of antioxidant enzymes activities	At 100 mg/kg: 16.3% for DPPH 56.9% for ABTS 1.00% for O_2_^−^ 17.8% for OH^−^ 2.63 mg/g protein for GSH 3.39 U/mg protein for GPx 54.0 U/mg protein for SOD 0.25 U/mg protein CAT	[19]
–	Purchased	0, 25, 75, and 100 mg/L Measurement of lipid peroxidation and hydrogen peroxide (H_2_O_2_) Semi-quantitative RT-PCR analysis	Reduced H_2_O_2_ generation Reduced ROS levels Up-regulated antioxidant enzyme (SOD, CAT, peroxydase (POD), and ascorbate peroxidase (APX)) activity Increased the content of carotene, ascorbic acid, and tocopherol	[20]
–	Not reported	Doses: 40, 60, 80, and 100 mg/kg for 42 days Determination of CAT, malondialdehyde (MDA), GSH, SOD, and GPx qRT-PCR analysis	Increased CAT activity in the jejunal mucosa Increased GSH content in the ileal mucosa	[18]
–	Purchased	Doses: 25 and 50 mg/kg Carbon tetrachloride (CCl_4_)–induced chronic liver injury in rats Immunohistochemistry	Increased intracellular enyzmic antioxidants (SOD and CAT) in rat liver tissue Reduced lipid peroxidation and fibrosis markers in rat liver tissue	[31]
–	Purchased	20-mg/kg p.o HFD-induced insulin resistance in gastrocnemius muscle Determination of ROS, LPO, and antioxidant enzymes Western blot analysis	Stabilized oxidative stress markers	[57]
Ergosterol	Not reported	^•^CH(OH)CH_3_ scavenging activity Density functional theory (DFT)	Scavenge efficiently the ^•^CH(OH)CH_3_ radical	[50]
–	Purchased	LPS-induced sepsis myocardial injury (in vivo and in vitro) MTT assay ELISA measurement Western blot analysis	Increased SOD activity Reduced MDA content Reduced LDH and CK-MB levels Restored HO-1 and Nrf-2 expression in rat hearts Inhibited cytochrome c	[42]
–	Purchased	CS-induced chronic obstructive pulmonary disease (COPD) model (in vivo and in vitro) 6HBE cells and Balb/c mice Measurement of SOD, MDA, and CAT production	Inhibited CS-induced oxidative stress by inhibiting NF-κB/p65 activation	[21]
–	*Coelastrella terrestris*	Doses: 12.5, 25, 50, 100, and 125 μg/mL DPPH assay	100% antioxidant effect achieved when used at 50 μg/mL	[59]
–	Purchased	Isoproterenol (ISO)-induced myocardial cardiotoxicity Hypoxia- reoxygenation model in H9C2 cells Western blot analysis	Decreased myocardial LDH and CK-MB levels Restored HO-1 and Nrf2 expression Inhibited cytochrome c	[54]
–	*Monascus anka*	Determination of lipid peroxidation inhibition rate PCS-201–012 cells Determination of cell proliferation Determination of ROS generation	Lipid peroxide inhibition rates at 2 μg/mL = 57.42% Reduced intracellular ROS level in damaged cells Improved survival rates of H_2_O_2_-induced damaged cells: - 43.88% at 200 mg/mL - 46.64% at 400 mg/mL	[43]
–	Purchased	Oxidation effect evaluation on plasma membrane integrity and yeast viability Lipid peroxidation evaluation DPPH assay	Involved in yeast resistance to *tert*-butyl hydroperoxide Protected lipids against oxidation in liposomes	[49]
Stigmasterol	*Butea monosperma*	Dose: 2.6 mg/kg p.o. for 20 days Swiss albino mice	Decreased hepatic lipid peroxidation (LPO) Increased CAT, SOD, and GSH activities	[40]
–	Purchased	Doses: 200 and 400 ppm Activity measurement of two enzymes involved in cell antioxidant defense system	Increased ascorbate peroxidase (APX) and glutathione reductase (GR) activities	[39]
–	Purchased	Antioxidant system analysis	Increased the level of antioxidant system components (CAT, GR, and APX) Reduced the adverse effects of salt stress on faba bean plants Reduced MDA and GSH contents	[36]
–	Purchased	Doses: 0, 0.2, 0.5, 1.0, 1.5, and 2 g/L MDA analysis Enzyme extraction and activity assay: polyphenol oxidase (PPO), CAT, SOD, and POD	At 0.5 g/L: Maintained at a higher level the activities of CAT and SOD Reduced POD activity Decreased MDA content Decreased PPO activity	[32]
–	*Grewia carpinifolia*	Dose: 100 μg Mice hippocampal homogenate Measurement of CAT, SOD, H_2_O_2_, and MDA expression (in vivo) Immunohistochemistry	Increased antioxidant enzyme activities Decreased oxidative stress markers and lipid peroxidation	[28]
–	Purchased	Doses: 20, 40, and 80 mg/kg Male Wistar rats Western blot analysis Oxidative stress biomarker quantification	Restored antioxidant defense system levels (Mn-SOD, T-SOD, GPX, and GSH)	[21]
–	Not reported	Doses: 20, 40, and 80 mg/kg p.o Stroke Wistar rats Antioxidant index determination Western blot analysis	Reduced MDA content Increased SOD, GSH, and GPx levels Activated Nrf2 signaling pathway	[38]
–	Not reported	Stable transfection Ishikawa cell line Immunohistochemistry staining Western blot analysis CCK8 assay	Activated Nrf2 signaling pathway	[53]
–	*Prangos ferulacea*	DPPH assay	Moderate antioxidant potential	[60]
–	Not reported	RAW264.7 macrophages Splenocytes MTT assay qRT-PCR analysis Western blot analysis	Increased NO production levels Increased HO-1 and iNOS expression levels Increased SOD activity in spleen	[34]
–	Not reported	100 ppm Determination of H_2_O_2_ and lipid peroxides Determination of GSH and POD Assay of antioxidant enzymes activity (CAT, GPX, APX, and GR)	Decreased lipid peroxidation and H_2_O_2_ Inhibited CAT, GPX, APX, and GR activities	[46]
–	Purchased	Doses: 100, 200, and 300 mg/L Sunflower plants grown under drought stress	Decreased MDA, H_2_O_2_, and lipoxygenase (LOX) enzyme Increased the levels of antioxidant enzymes (GR, CAT, SOD, POX, etc.)	[35]
Fucosterol	*Pelvetia siliquosa*	CCI_4_-intoxication in rats	Increased the activities of anti-oxidant enzymes SOD (33.89%), CAT (21.56%), and GPX (39.24%)	[37]
–	*Padina boryana*	PM-induced inflammation in RAW264.7 macrophages MTT assay Western blot analysis qRT-PCR analysis	Activated Nrf2/HO-1 signaling pathway	[52]
–	*Sargassum horneri*	Human dermal fibroblast (HDF) MTT assay 2’,7’-dichlorofluorescein diacetate (DCF-DA) assay Western blot analysis RT-PCR analysis	Activated Nrf2/HO-1 signaling pathway	[25]

Regarding cellular antioxidant mechanisms, numerous investigations have shown that phytosterols are able to activate antioxidant enzymes to reduce the production of ROS and prevent oxidative damage. Indeed, *β*-sitosterol, fucosterol, stigmasterol, and ergosterol all induced the activation of multiple antioxidant enzymes, namely CAT, SOD, GSH, glutathione peroxidase (GPx), glutathione reductase (GR), ascorbate peroxidase (APX), and ceruloplasmin [19–21, 28–42]. Additionally, stigmasterol restored the levels of two types of SOD present in living cells; Mn-SOD (mitochondrial SOD) and T-SOD (extracellular SOD) [21]. Moreover, levels of certain non-enzymatic antioxidants such as tocopherol, ascorbic acid, carotene, and vitamins C and E were also increased by *β*-sitosterol [20, 33]. On the other hand, the activity of polyphenol oxidase (PPO) and peroxidase (POD), two types of enzymes that catalyze oxidation reactions and are commonly present in plants, was up-regulated by *β*-sitosterol [20] and stigmasterol [35].

Importantly, stigmasterol exhibited antioxidant activity by decreasing the adverse effects of salt stress on plants (bean) [36]. This stress, of environmental origin, can accumulate free radicals in plants, which can have negative effects on crop yield and growth. Antioxidants, by reducing oxidative damage, can increase cell survival in response to elevated free radical levels. This was achieved by ergosterol, which improved H_2_O_2_-induced damaged cells’ survival rates [43]. Paradoxically, a balanced production of nitric oxide (NO) can also have antioxidant effects under certain conditions, as recently demonstrated by stigmasterol [34].

**Fig. 2:**
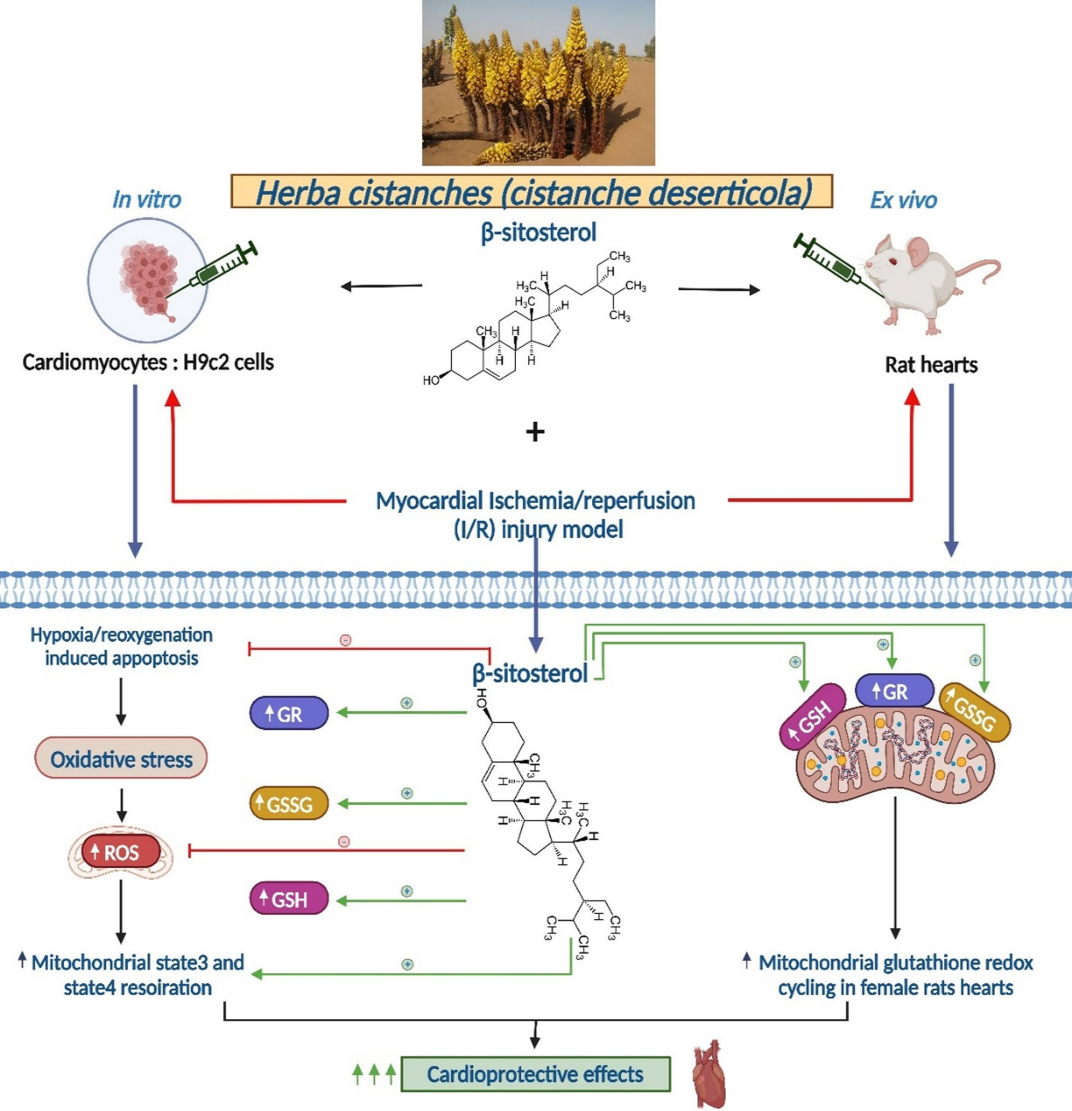
Potential mechanistic pathways of the antioxidant effect of *β*-sitosterol leading to cardioprotective benefits using in vitro and ex vivo experiments. Mitochondrial ROS generation was enhanced by *β*-sitosterol. This phytosterol also increased mitochondrial respiration in states 3 and 4, decreasing coupling efficiency. In H9c2 cells, *β*-sitosterol increased glutathione redox cycling (GR, GSH, and GSSG) and protected against hypoxia/reoxygenation-induced apoptosis. Ex vivo, *β*-sitosterol protected the myocardium against I/R injury in female rats. The cardioprotective effect of *β*-sitosterol in this category was most likely mediated by an increase in mitochondrial glutathione redox cycling (GR, GSH, and GSSG). GR: glutathione reductase; GSH: reduced glutathione; GSSG: oxidized glutathione; ROS: reactive oxygen species; I/R: ischemia/reperfusion

For subcellular mechanisms, *β*-sitosterol stimulated mitochondrial ATP-producing capacity in H9c2 cells [44] (Fig. 2). Low ATP production can impair mitochondrial functions, leading to oxidative stress. Maintaining the balance between mitochondrial ATP production and mitochondria protection against oxidative damage is therefore very important.

Moreover, inhibition of lipid peroxidation (LP), a lipid oxidation process in cell membranes, has emerged as a crucial subcellular defense mechanism against oxidative stress. *β*-sitosterol, ergosterol, and stigmasterol either prevented LP alterations or reduced levels of lipid peroxidative products [28, 31, 40, 43, 45, 46]. In this sense, *β*-sitosterol decreased the levels of thiobarbituric acid reactive substances (TBARS) [33, 47], often used as markers of LP. By inhibiting LP, *β*-sitosterol thus has the ability to reduce TBARS levels, which may ultimately protect tissues and cells from oxidative damage. The antioxidant effectiveness of *β*-sitosterol is illustrated by the reduction of TBARS levels. The content of another LP by-product used as a marker of cell damage and oxidative stress called malondialdehyde (MDA) has been strongly inhibited by stigmasterol and ergosterol in several studies [32, 35, 36, 38, 42]. Modulation of cell signaling may also constitute another subcellular antioxidant mechanism, namely the modulation of signaling pathways involved in programmed cell death, such as the apoptosis pathway. *β*-Sitosterol is an interesting molecule in this area, as it has been shown to protect H9c2 cells against hypoxia/reoxygenation-induced apoptosis [44] and to prevent GOX-induced oxidative stress and LP via estrogen receptor (ER)-mediated PI3K/GSK3*β* signaling [48].

Antioxidants can protect lipids from oxidation in liposomes, structures used for transporting drugs or other substances. However, the lipids that form liposomes are very vulnerable to oxidation, which can affect their effectiveness as drug carriers. This is why it is imperative to protect lipids against oxidation to maintain liposome stability. This mechanism was observed in the study carried out by Dupont et al. [49] using ergosterol.

At the molecular level, several antioxidant mechanisms have been demonstrated. Free radical protection in cell membranes has been a promising therapeutic approach in neutralizing free radicals before they can cause damage. Indeed, stigmasterol, ergosterol, and *β*-sitosterol reduced the generation and levels of several radicals such as DPPH, ABTS, H_2_O_2_, O_2_^⋅−^, OH^−^, NO, hydroperoxides (HPO), and ^•^CH(OH)CH_3_, as well as that of an enzyme catalyzing free radical formation, lipoxygenase (LOX) [19, 20, 33, 35, 41, 46, 47, 50]. In addition, *β*-sitosterol [20] and ergosterol [43] showed a significant reduction in intracellular ROS levels. This reduction in ROS is essential to protect cells against oxidative damage. In fact, overproduction of ROS can lead to cellular damage, whereas these phytosterols can neutralize ROS and avoid their harmful effect, as suggested by previous results. By regulating the redox balance, they can also maintain cellular health and prevent possible diseases. This was achieved by *β*-sitosterol which up-regulated the redox cycle of cellular glutathione [44].

In contrast, activation of Nrf2 and Nrf2/HO-1 signaling pathways can be used against oxidative stress. It has been shown that during cellular stress, these pathways are activated, and as a result the protein called nuclear factor-erythroid 2-related factor 2 (Nrf2) present in the cell cytoplasm moves to the nucleus and activates gene expression involved in toxic substance detoxification, DNA repair, and defense against oxidative stress [51]. Recently, it was discovered that stigmasterol, ergosterol, and fucosterol are able to activate these two pathways, allowing them to be effective natural compounds in oxidative stress management [25, 38, 42, 52–54].

It was indicated in the previous section that the protein NF-κB/p65 plays an important role in inflammation and immune response, but an over-activation of this protein can increase the generation of free radicals and increase OS in cells. This indicates that inhibition of its activation is an additional antioxidant mechanism at the molecular level. Indeed, Sun et al. [21] recorded a decrease in CS-induced OS through the inhibition of NF-κB/p65 activation by administering ergosterol.

This phytosterol also inhibited cytochrome c [42, 54], a protein involved in the electron transport chain of cellular respiration. However, excess electron production can lead to the formation of free radicals. In this context, cytochrome c inhibition may be an optional strategy to protect cells from free radical damage and prevent cellular oxidation.

Moreover, several investigations have found that decreased OS markers may constitute a crucial molecular antioxidant mechanism [55, 56]. In our context, *β*-sitosterol and stigmasterol reduced and stabilized oxidative stress markers in different tissues (in vivo/in vitro) [28, 31, 57], whereas ergosterol reduced the levels of two biomarkers, namely lactate dehydrogenase (LDH) and creatine kinase MB (CK-MB) [42, 54], used to assess cell damage as they may also be overexpressed in various conditions, including CVDs.

As previously demonstrated, stigmasterol has antioxidant potential by stimulating the production of NO that can act as an antioxidant [34]. In the same study, authors highlighted a molecular antioxidant effect related to increased expression levels of the iNOS enzyme, which catalyzes the synthesis of NO. It is therefore clear that increased NO production is closely dependent on iNOS expression. However, it should be noted that the mechanisms regulating the expression of this enzyme can be complex and depend on many factors.

### Anti-inflammatory mechanisms

Several investigations have examined the anti-inflammatory potential of various phytosterols by shedding light on the mechanisms of action. Indeed, the majority of these studies have shown that these natural compounds exert their anti-inflammatory effects at various levels via cellular, subcellular, and molecular mechanisms.

At the cellular level, several phytosterols inhibited experimentally induced edema in animals, in particular paw and ear edema, such as *β*-sitosterol [61–64], stigmasterol [65–67], and ergosterol [68]. In addition to the reduction in paw edema recorded by Zhang et al. [64] with *β*-sitosterol, a decrease in the polyarthritic index used to assess the severity of joint damage in subjects with polyarticular forms of arthritis. It has been shown that in rheumatoid arthritis, the immune system attacks various body tissues, particularly joints, subsequently inducing chronic inflammation.

Additionally, *β*-sitosterol and stigmasterol showed notable anti-inflammatory effects (in vivo) against colonic inflammation (colitis) by alleviating its severity [69–71] and score [69] or inhibiting colon shortening [72] that is a major consequence of inflammation.

On the other hand, a powerful anti-inflammatory activity was obtained by *β*-sitosterol, which stimulated the secretion of certain molecules involved in inflammatory processes, namely histamine, bradykinin, serotonin, and prostaglandins [62].

Another mechanism associated with an inflammatory immune response has been observed with this phytosterol, which is the increase in calcium absorption in activated neutrophils [73]. Activation of these white blood cells (neutrophils) in response to tissue damage or infection, triggers biochemical reactions to produce free radicals and inflammatory enzymes that target damaged cells and pathogens [74]. In activated neutrophils, the production of these enzymes is closely linked to the absorption of calcium having the role of a cellular messenger [75]. This makes neutrophils indispensable immune cells in inflammatory responses by migrating to the site of inflammation to destroy pathogens and remove damaged structures. However, chronic inflammation and further tissue damage may occur as a result of an excessive inflammatory reaction via an immunopathological process [76]. For this reason, inhibiting neutrophil migration/recruitment may provide more benefit in certain inflammatory processes. This was the aim of two very recent studies conducted by Santos et al. [77] and Zhang et al. [78] who recorded an inhibitory effect on the recruitment and migration of these cells, respectively, by ergosterol and *β*-sitosterol. As with chronic/excessive inflammatory responses, tissue damage may occur, and leukocyte infiltration and microglial activation that are normal body responses to infection may, in turn, contribute to the development of various diseases in chronic conditions. Therefore, reducing leukocyte over-infiltration and microglial over-activation will be promising therapeutic option in inflammation. Indeed, stigmasterol [67] and ergosterol [26] remarkably inhibited leukocyte infiltration and microglial activation, respectively, using different methods.

Moreover, all the molecules presented in Table 2 (*β*-sitosterol, stigmasterol, ergosterol, and fucosterol) have inhibited the expression of the inducible nitric oxide synthase (iNOS) [22, 23, 52, 79–82], which catalyzes the synthesis of nitrogen monoxide (NO) involved in the regulation of inflammation in immune cells [83]. It has often been shown that the expression of iNOS is induced by pro-inflammatory cytokines such as interleukin-1 (IL-1), IL-6, and tumor necrosis factor-α (TNF-α) [84]. In addition, the NO synthesis induced by iNOS can cause tissue damage and chronic inflammation, particularly ulcerative colitis and rheumatoid arthritis. This makes the inhibition of iNOS expression a colossal anti-inflammatory mechanism. Furthermore, *β*-sitosterol alone up-regulated the level of endothelial NOS (eNOS) [85], which is another type of NOS catalyzing the production of NO from arginine.

**Table 2:** Anti-inflammatory properties of phytosterols

Molecules	Origins	Experimental methods	Key findings	Refs.
*β*-sitosterol	*Ranunculus sceleratus* L	MTT assay 5-lipoxygenase (5-LOX) activity Oxazolone-induced mouse ear oedema	Reduced oedema (in vivo) No effect on arachidonate pathway enzymes implicated in the inflammatory process (in vitro)	[63]
–	Purchased	Human aortic endothelial cells (HAECs) Monocyte (U937 cells) Attachment in tumor necrosis factor-α (TNF-α)-stimulated HAECs MTT assay Cytokine quantitation: ELISA	Inhibited intracellular adhesion molecule 1 (ICAM-1) and vascular cell adhesion molecule 1 (VCAM-1) expression in TNF-α Inhibited U937 cell binding to TNF-α Attenuated nuclear factor-κB (NF-κB) p65 phosphorylation	[90]
–	*Nyctanthes arbortristis*	Doses: 5, 10, and 20 mg/kg, i.p Wistar rats Hind paw oedema	Inhibited paw oedema	[110]
–	*Trachelospermum jasminoides*	Lipopolysaccharide (LPS)-stimulated RAW264.7 murine macrophages	Inhibited the activities of interleukin-6 (IL-6) of stimulated macrophages Reduced TNF-α and IL-1*β* secretion	[111]
–	Purchased	2,4,6-trinitrobenzene sulfonic acid (TNBS)-induced colitis in mice C57BL/6 mice Immunoblotting ELISA measurement	Decreased myeloperoxidase (MPO) activity (in vivo) Attenuated NF-κB Inhibited inflammatory enzymes Inhibited pro-inflammatory cytokines	[72]
–	*Esenbeckialeiocarpa*	Carrageenan-induced inflammation in the mouse air pouch model Calcium uptake measurement MPO and adenosine-deaminase (ADA) activity quantification	Increased calcium uptake in activated neutrophils Inhibited ADA and MPO activity Inhibited TNF-α and IL-1*β* levels	[73]
–	Purchased	Mice with colitis after being fed a high fat diet (HFD) Intestinal mouse macrophages stimulated with lipopolysaccharides (LPS) ELISA measurement Immune blot analysis	Ameliorated HFD-induced colitis through inhibition of LPS binding to toll-like receptor 4 (TLR4) in the NF-κB pathway	[71]
–	*Justicia gendarussa* burm. F	Carrageenan-induced rat paw oedema model Calculated the percentage inhibition of oedema	Induced potent anti-inflammatory effect by secreting prostaglandin (69.43%), serotin and bradykinin (52.25%), and histamine (30.07%)	[62]
–	Purchased	26 C57BL/6 J male mice were fed a high fat western-style diet (HFWSD) and subsequently developed colitis through the induction of dextran sulfate sodium (DSS)	Reduced the severity of colitis Suppressed NF-κB activation Decreased colonic inflammation score Decreased COX-2 expression	[69]
–	*Oxalis corniculata*	Doses: 5, 10, and 20 mg/kg, i.p Rat paw oedema model	After 120 min, the administration of a dosage of 20 mg/kg resulted in: The inhibition of rat paw edema, reducing it to a volume of 0.32 ± 0.06 mL	[61]
–	*Moringa oleifera*	Doses: 7.5 − 30 μM HaCaT cells (keratinocytes) J774A.1 cells (macrophages)	Secretion of inflammatory factors (IL-1*β*, IL-6, TNF-α, and ROS) from both keratinocytes and macrophages was effectively suppressed Inhibited caspase-1 activation Partially inhibited NF-κB in macrophages	[101]
–	Purchased	Wistar male rats ELISA measurement RT-PCR analysis	Inhibiting inflammatory reactions, particularly TNF-α	[112]
–	Purchased	LPS-exposed BV2 murine microglial cells CCK-8 assay ELISA measurement RT-PCR analysis Western blot analysis	Reduced inflammatory mediator [IL-6, COX-2, TNF-α, and inducible nitric oxide (iNOS)] expression Inhibited NF-κB, ERK, and p38 pathway activation	[80]
–	Purchased	Induction of arthritis via complete freund’s adjuvant Swiss Wistar rats Poly-arthritic index ELISA measurement qRT-PCR analysis	Reduced paw edema and arthritic index Reduced cytokines Reduced the level of COX-2, prostaglandin E_2_ (PGE_2_), NF-κB, and vascular endothelial growth factor (VEGF)	[64]
–	Purchased	Dose: 1 mg/kg Puncture (CLP)-induced septic rats ELISA measurement RT-qPCR analysis	Decreased cytokine levels	[97]
–	Not reported	Dose: 150 mg/kg, p.o Female Wistar rats ELISA measurement	Down-regulated IL-6 and TNF-α levels Up-regulated endothelial nitric oxide synthase (eNOS) level	[85]
–	Purchased	RAW264.7 cells were stimulated with LPS, while C57BL/6 J mice developed acute lung injury as a result of LPS induction ELISA measurement RT-PCR analysis Western blot analysis	Suppressed the mRNA levels of IL-6, TNF-α, and IL-1*β* (in vitro) Inhibited the activation of TLR4/NF-κB pathway activation	[108]
–	Purchased	A zebrafish larvae (*Danio rerio*) model of inflammation Neutrophil migration assay	Inhibited neutrophil migration to the lateral line Reduced inflammatory gene (*myd88* and *il-8*) expressions	[78]
Ergosterol	*Sarcodon aspratus* (Berk.) S. Ito	Murine macrophages (RAW264.7) HT29 colorectal adenocarcinoma cells	Inhibited TNF-α secretion Inhibited IL-1α/*β* expression Suppressed JNK, MAPK p38, and ERK phosphorylation	[98]
–	Purchased	LPS-induced inflammation in RAW264.7 macrophages MTT assay Flow cytometry analysis Western blotting analysis	Down-regulated proteins associated with the NF-κB cascade Inhibited TNF-α production Inhibited COX-2 expression	[99]
–	*Cordyceps militaris*	BV2 microglia cells	Reduced nitric oxide (NO) in LPS triggered BV2 cells	[87]
–	*Scleroderma Polyrhizum* Pers.,	Doses: 25 and 50 mg/kg LPS-induced acute lung injury	Inhibited inflammatory cells Inhibited pro-inflammatory cytokines (TNF-α and IL-6) Inhibited NF-κB, COX-2, and iNOS activation	[104]
–	Purchased	Chronic obstructive pulmonary disease (in vivo) ELISA measurement Western Blot analysis	Inhibited cytokines in lung and serum Inhibited expression of JAK3/STAT3/NF-κB pathway (in vivo)	[95]
–	*Ganoderma sinense*	NO production in RAW264.7 macrophages	Exhibited anti-inflammatory activities against NO production IC_50_ = 17.7–32.4 μM	[86]
–	Purchased	CS-induced chronic obstructive pulmonary disease (COPD) model (in vivo and in vitro) 6HBE cells and Balb/c mice ELISA measurement Western blot analysis NF-κB/p65 activity assay	Inhibited CS-induced o inflammation by inhibiting NF-κB/p65 activation	[21]
–	*Anvillea garcinii*	5-LOX inhibitory potential	Displayed moderate 5-LOX inhibitory activity (IC_50_ = 3.06 − 3.57 mM)	[107]
–	Purchased	Diabetic nephropathy-induced inflammation (in vivo) ELISA measurement RT-PCR analysis Western blot analysis	Decreased inflammatory cytokine (MCP-1, TNF-α, and IL-6) levels Decreased NF-κB signaling pathway	[102]
–	*Cryptoporus volvatus*	LLC-PK1 cells RT-PCR analysis Western blot analysis Immunofluorescence assay Flow cytometry assay	Decreased the mRNA levels of cytokines (PKR, Mx1, IFN-*β*, IFN-α)	[92]
–	*Sclerotinia Sclerotiorum*	Croton oil-induced ear edema quantification of MPO enzyme	Inhibited ear edema, via inhibition of COX pathway activity Inhibited neutrophil recruitment	[77]
–	Purchased	TNF-α-induced HT-22 hippocampal cell injury Western blot analysis RT-PCR analysis	Attenuated TNF-α toxicity on HT-22 cells Induced the overexpression of the EGR-1 transcription factor	[113]
–	*Antrodia camphorata*	LPS-induced microglial activation and neuro-inflammatory reactions (in vivo and in vitro) BV2 and HMC3 microglial cells Male ICR mice Western blot analysis Immunohistochemistry RT-PCR analysis	Inhibited protein kinase B (AKT), NF-κB, and mitogen-activated protein kinase (MAPK) signaling pathways (in vitro) Decreased the levels of NF-κB phosphorylation and cytokines (in vivo)	[26]
Stigmasterol	*Mondia whytei*	Doses: 7.5, 15, 30, and 100 mg/kg Dimethylbenzene-induced ear edema test Adult Swiss albino mice	Inhibited ear edema Induced anti-inflammatory activity (50.34% at 30 mg/kg)	[66]
–	Purchased	Human umbilical vein endothelial cells MTT assay Western blot analysis RT-PCR analysis Immunohistochemistry	Down-regulated TNF-α No effect on other cytokines (IL-6 and CXCL-8)	[96]
–	Purchased	Colitis in 26 C57BL/6 J male mice fed a HFWSD	Reduced the severity of colitis Suppressed NF-κB activation Decreased colonic inflammation score Decreased COX-2 expression	[69]
–	Purchased	Irritant dermatitis	Controlled inflammatory characteristics (neutrophilia and ear skin edema) Suppressed pro-inflammatory cytokine expression Reduced TNF-α serum levels	[65]
–	Purchased	Collagen-induced arthritis (CIA)-induced inflammation in rats ELISA measurement qPCR analysis	Inhibited TNF-α/IL-6/IL-1*β*/iNOS/COX-2 expression Augmented anti-inflammatory cytokine IL-10 expression Down-regulated NF-kBp65/p38MAPK expression Inhibited p-IKB-α activation	[22]
–	Purchased	Peritonitis Paw edema	Reduced paw edema Decreased leukocyte infiltration Prevented paw licking Presented anti-inflammatory effects	[67]
–	Purchased	IL-1*β*-induced inflammation in primary rat articular chondrocytes MTT assay qRT-PCR analysis Western blot analysis	Reduced IL-6 and iNOS expression Stigmasterol + mesenchymal stem cells-condition medium (MSC-CM) normalized pro-inflammatory/pro-catabolic responses and inhibited NF-κB activation	[23]
–	*Petiveria alliacea*	Pro-inflammatory mediator release by egg albumin protein Molecular Docking Studies against TNF-α and COX-2	At 6.5 kcal/mol: Better binding energy against TNF-α At 9.7 kcal/mol: Identified as hit molecule against COX-2 enzyme	[114]
–	Purchased	APP_swe_/PS1_dE9_ mice Simulated BV2 cells with A*β*_42_ oligomers ELISA measurement CCK8 assay Flow cytometry assay Western blot analysis	Reduced microglia activation Reduced pro-inflammatory cytokine levels Protected BV2 cells via AMPK/NLRP3 and AMPK/NF-κB signaling	[24]
–	Not reported	RAW264.7 macrophages Splenocytes MTT assay qRT-PCR analysis Western blot analysis ELISA measurement	Increased IL-2 and IFN-γ levels in RAW264.7 cells Up-regulated NF-κB activation in RAW264.7 cells Increased TNF-α secretion levels in splenocytes	[34]
Fucosterol	*Undaria pinnatifida*	LPS-induced RAW264.7 macrophages Western blot analysis qRT-PCR analysis	Inhibited iNOS, TNF-α, and IL-6 expression Suppressed p38 MAPK and NF-κB pathways	[81]
–	*Eisenia bicyclis*	LPS-induced RAW264.7 macrophages MTT assay	Suppressed COX-2 and iNOS expression Suppressed NF-κB pathway	[79]
–	*Hizikia fusiformis*	Atopic dermatitis-like lesions (in vivo) Mouse macrophage cells RAW264.7 macrophages Rat basophilic leukemia RBL-2H3 cells ELISA measurement	Decreased LPS-induced production of NO (in vitro) Decreased DNCB-stimulated serum immunoglobulin E (IgE) levels Inhibited TNF-α and IL-4 levels Increased IFN-γ secretion	[88]
–	Purchased	Acute lung injury (in vivo) Alveolar macrophages (in vitro) MTT assay ELISA measurement Western blot analysis	Attenuated cytokine production (in vivo) Inhibited NF-κB activation and cytokine production (in vitro)	[100]
–	*Hizikia fusiformis*	Hypoxia damages to keratinocytes (HaCaT)	Demonstrated a dose-dependent suppression of inflammation Inhibited the expression of IL-1*β*, IL-6, and TNF-α in HaCaT cells Suppressed HIF1-α accumulation	[103]
–	Purchased	*β*-amyloid (A*β*)- and LPS-induced microglial cells ELISA measurement RT-PCR analysis	Inhibited IL-1*β*, IL-6, TNF-α, PGE_2_, and NO production	[89]
–	*Sargassum binderi*	Particulate matter (PM)-induced inflammation in A459 human lung epithelial cells Western blot analysis	Suppressed COX-2 and PGE_2_ Inhibited TNF-α and IL-6 Suppressed MAPK and NF-κB pathways	[93]
–	*Padina boryana*	PM-induced inflammation in RAW264.7 macrophages MTT assay Western blot analysis qRT-PCR analysis	Down-regulated dose-dependently the production of inflammatory mediators (PGE_2_, COX-2, and iNOS) and pro-inflammatory cytokines (TNF-α, IL-6, and IL-1*β*) Suppressed NF-κB and MAPK pathways	[52]
–	*Sargassum horneri*	Human dermal fibroblast (HDF) Western blot analysis RT-PCR analysis	Down-regulated inflammatory mediators Regulated Nrf2/HO-1 signaling Regulated NF-κB and MAPK signalling	[25]

In this sense, the inhibition of NO production has been observed in vitro by ergosterol [86, 87] and fucosterol [88, 89] on two different types of immune cells, RAW264.7 macrophages and microglial cells with similarities in responses and functions.

It has been shown above that phytosterols can inhibit neutrophil migration/recruitment, and to better understand the underlying molecular mechanisms several studies have been performed. At the molecular level, one of the most common and oldest approaches is to target adhesion molecules (AM) involved in the migration of neutrophils to the inflammation sites. In 2010, Loizou and colleagues examined this attachment in human aortic endothelial cells stimulated with TNF-α after *β*-sitosterol treatment [90]. The results showed an inhibition of the expression of two AMs, namely vascular cell AM-1 (VCAM-1) and intracellular AM-1 (ICAM-1). Through these molecules, neutrophils can attach to endothelial cells in the blood vessel wall and then migrate to the surrounding tissues. Drugs that block the expression of these AMs may thus inhibit neutrophil migration and consequently reduce inflammation.

Another therapeutic approach is to target pro-inflammatory cytokines responsible for recruiting neutrophils to sites of inflammation. Several authors have targeted the inhibition of cytokine activities or their mRNA levels as an anti-inflammatory strategy. Whether in vitro or in vivo, all the phytosterols already mentioned showed remarkable inhibitory potential in the expression of pro-inflammatory cytokines (IL-2, IL-4, IL-6, IL-8, IL-10, IL-12, IL-13, IL-16, IL-17, IL-33, IL-1*β*, IL-1α/*β*, TNF-α, IFN-α, IFN-*β*, etc.) [23–26, 52, 64, 72, 73, 80, 81, 85, 88, 89, 91–104]. In fact, by activating specific receptors on neutrophils, certain cytokines like IL-8 (CXCL-8) direct these cells to inflammatory sites, and targeting these cytokines can attenuate neutrophil infiltration into inflammatory tissues.

In contrast, overexpression of certain proteins associated with immune and inflammatory responses may also contribute to the development of chronic inflammatory diseases such as multiple sclerosis and rheumatoid arthritis. At this molecular level, ergosterol decreased levels of Myxovirus Resistance protein 1 (Mx1), Protein Kinase R (PKR), Monocyte Chemoattractant Protein-1 (MCP-1), and Ionized calcium-Binding Adapter molecule 1 (IBA-1) [26, 92, 102]. In general, Mx1 and PKR are two proteins involved in the antiviral response and are expressed in certain inflammatory tissues and immune cells, whereas MCP-1 is responsible for the recruitment of macrophages and monocytes in inflammatory tissues, and IBA-1 is expressed mainly in microglial cells. However, hyper-activation of some of these molecules can lead to inflammation by activating the secretion of inflammatory cytokines. Regulation of these proteins is therefore important in maintaining the balance between excessive inflammatory damage and the protective immune response.

Furthermore, *β*-sitosterol reduced the expression of the NOD-like receptor family, pyrin domain containing 3 (NLRP3), a key component of the inflammasome that is involved in the innate immune response [101]. Activation of the NLRP3 inflammasome is mediated by various stimuli (cellular stress, tissue damage, infections, etc.) and stimulates the production of IL-18 and IL-1*β*. In this case, the decrease in the expression of NLRP3 can inhibit the production of these cytokines and exhibit an anti-inflammatory effect. Additionally, activation and maturation of these pro-inflammatory cytokines (IL-18 and IL-1*β*) are associated with the involvement of an inflammatory enzyme called caspase-1, which in hyper-activation can cause chronic inflammatory diseases. The discovery of new caspase-1 inhibitors can attenuate tissue damage and reduce inflammation, which has been seen with *β*-sitosterol (in vitro) [101]. This molecule has also been shown to reduce the activity of myeloperoxidase (MPO) [72, 73], an inflammatory enzyme secreted by immune cells of the innate immune system (macrophages, monocytes, and neutrophils). Despite this beneficial potential, excessive activity or high levels of this enzyme can lead to CVDs and chronic inflammatory processes. In addition to this anti-inflammatory mechanism, the same study carried out by Liz et al. [73] noted the inhibition of an enzyme called adenosine-deaminase (ADA), which degrades adenosine related to inflammation regulation. This indicates that inhibiting ADA activity can potentially attenuate inflammation.

Moreover, *β*-sitosterol inhibited the expression of another inflammatory enzyme, cyclooxygenase (COX)-2 [64, 72], also induced by cytokines as well as other inflammatory stimuli (pathogens, hormones, and growth factors). Similarly, ergosterol inhibited the activity of the COX pathway [77], an important metabolic pathway related to the synthesis of prostaglandins (PGE_2_), molecules highly involved in inflammatory processes. The production of these chemical mediators of inflammation (PGE_2_) was further inhibited by *β*-sitosterol in the same study performed by Zhang et al. [64] as well as by fucosterol in several in vitro investigations [52, 89, 93].

Another protein responsible for angiogenesis and vascular regeneration may act as an inflammatory mediator, called vascular endothelial growth factor (VEGF), by increasing vascular permeability and recruitment of inflammatory cells to sites of inflammation. Adopting this strategy, *β*-sitosterol [64] and stigmasterol [96] reduced VEGF signaling levels in vivo and in vitro, respectively. Interestingly, *β*-sitosterol reduced these levels from 53.95 ± 2.90 to 11.68 ± 1.14 pg/mL compared to the control group.

On the other hand, fucosterol reduced serum levels of immunoglobulin E (IgE) which is one of the immune system antibodies [88]. Indeed, increasing serum IgE levels can induce inflammatory processes. In addition, inhibition of the activity of 5-lipoxygenase (5-LOX), an enzyme that synthesizes inflammatory mediators, has been proposed as an effective strategy in reducing inflammation and treating many diseases, in particular CVDs. Several natural compounds have been identified as inhibitors of this enzyme activity [105, 106], including phytosterols (*β*-sitosterol and ergosterol) [63, 107].

In addition to the normalization of pro-inflammatory responses already recorded with various phytosterols, the normalization of pro-catabolic responses has recently been observed with stigmasterol [23], since chronic inflammation can increase pro-catabolic processes. In fact, pro-catabolic responses are normal metabolic processes responsible for energy production through nutrient release and body tissue breakdown that are essential in maintaining homeostasis. However, an exaggerated increase in pro-catabolic responses can lead to certain negative effects.

Very recently, Zhang et al. [78] showed that *β*-sitosterol decreases the expressions of inflammatory genes (*myd88* and *il-8*), which can occur in response to multiple factors, namely toxins, injuries, and infections.

Importantly, regulation of the expression of these genes occurs through signaling pathways by turning on/off specific genes. The NF-κB signaling pathway, in particular, is a pathway that regulates the expression of several inflammatory genes and is activated in response to various stimuli (inflammatory cytokines, oxidative stresses (OSs), and infections), which can cause chronic inflammation in extreme situations (over-activation). Thus inhibiting the activation of this pathway will be an interesting anti-inflammatory approach. The four phytosterols presented in Table 2 all inhibited (in vivo and in vitro) the activation of the NF-κB signaling pathway [23, 25, 26, 52, 64, 69, 72, 79–81, 92, 93, 96, 97, 99–102, 104].

A protein belonging to the family of this pathway, NF-κB/p65, when activated can excessively produce pro-inflammatory cytokines. Activation of this subunit of the NF-κB transcription factor complex (NF-κB/p65) was inhibited by certain phytosterols, namely *β*-sitosterol [90], ergosterol [21], and stigmasterol [22].

In 2020, two studies showed that in addition to inhibiting NF-κB pathway activation, *β*-sitosterol can inhibit other signaling pathways as well as transcription factors involved in various cellular processes, including inflammatory reactions and immune responses [64, 80].

Indeed, Sun et al. [80] recorded activation inhibition of extracellular signal-regulated kinase (ERK) and p38 belonging to the family of mitogen-activated protein kinase (MAPK) signaling pathways. Both are activated in response to a variety of stimuli, especially inflammatory cytokines and once activated they phosphorylate downstream targets, including kinases and transcription factors to regulate inflammatory processes. While Zhang et al. [64] noted expression inhibition of receptor activator of NF-κB ligand (RANKL) (a TNF-family cytokine) and signal transducer and activator of transcription 3 (STAT3), a transcription factor activated in response to cytokines. Their overexpression has been implicated primarily in chronic inflammation. Similarly, two other studies obtained, with the same molecule, the inhibition of toll-like receptor 4 (TLR4)/NF-κB pathway activation; also involved in inflammation [71, 108]. When TLR4 detects a pathogen, it triggers a signaling cascade that activates the NF-κB pathway. However, like most of the aforementioned phenomena, prolonged activation of the TLR4/NF-κB pathway can lead to chronic inflammation. This makes this pathway a promising therapeutic target in the management of inflammatory diseases.

**Fig. 3:**
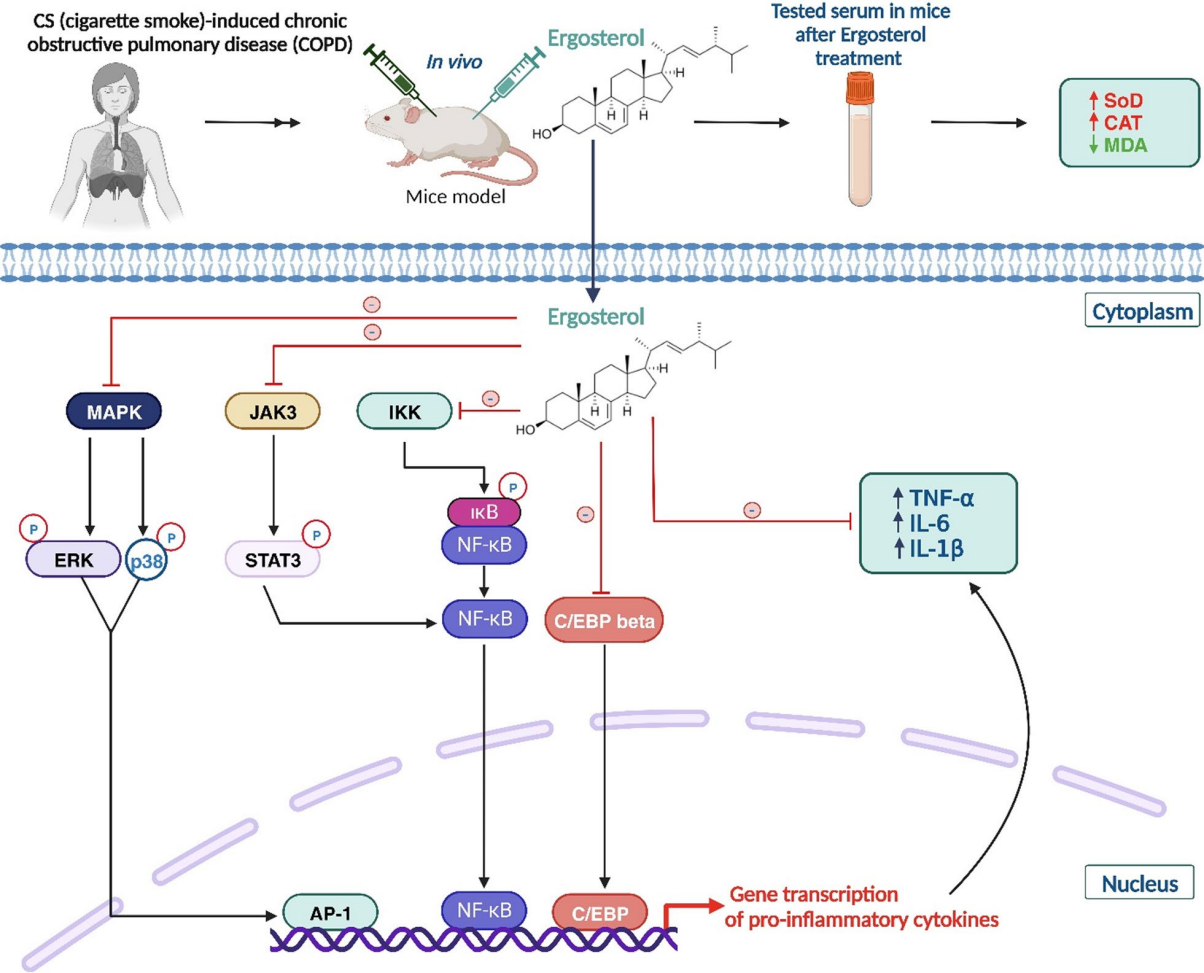
Illustration of the potential in vivo anti-inflammatory characteristics of available ergosterol on CS-induced COPD via the JAK3/STAT3/NF-κB signaling pathway. In CS-induced COPD, ergosterol phytosterol has been shown to inhibit phosphorylation of p38 MAPK, STAT3, NF-κB, and C/EBP protein expression. Consequently, when ergosterol was supplied, pro-inflammatory mediators such as IL-1 and IL-6, as well as TNF-α, were all less expressed in the in vivo experiment. In addition, ergosterol can effectively promote SOD and CAT activity in serum, while decreasing MDA content. ERK: extracellular signal-regulated kinase; MAPK: p38 mitogen-activated protein kinase; NF-κB: nuclear factor κB; AP-1: activating protein-1; STAT3: signal transducer and activator of transcription-3; IKK: IkB kinase; IkB: NF-kB inhibitor protein; IL: interleukin; C/EBP: CCAAT/enhancer binding protein; SOD: superoxide dismutase; CAT: catalase; MDA: malondialdehyde; CS-induced COPD: cigarette smoke-induced chronic obstructive pulmonary disease

Accordingly, ergosterol inhibited the protein expression of the JAK3/STAT3/NF-κB cell-signaling pathway [95], which plays an important role in regulating the inflammatory/immune response, while its dysregulation can develop inflammatory and autoimmune diseases. This process is cascading. When cytokines bind to the appropriate membrane receptors, Janus kinase 3 (JAK3), which is a kinase responsible for cytokine signal transduction, is activated and subsequently phosphorylates the transcription factor STAT3 that regulates gene expression. Then, the JAK3/STAT3 pathway can activate the NF-κB pathway inducing the expression of inflammatory genes and the production of pro-inflammatory cytokines. Moreover, this sterol inhibited the DNA-binding activity of NF-κB and also of another transcription factor involved in inflammation, C/EBP*β* [98]. In fact, in an inflammatory context, C/EBP*β* is activated by certain pro-inflammatory cytokines (TNF-α and IL-1), leading to the expression of genes involved in inflammation. In the same study, inhibition of p38, JNK, and ERK MAPK phosphorylation was recorded via the same molecule, which may subsequently reduce inflammation by reducing the production of pro-inflammatory cytokines and activation of immune cells (Fig. 3).

Similarly, stigmasterol down-regulated the expression of the p38/MAPK-signaling pathway and of NF-κBp65, a subunit of the transcription factor NF-κB [22], which in turn was inhibited by this molecule very recently in addition to protein kinase B (AKT), also known as protein kinase B-alpha (PKBα) [26]. This protein enzyme is known to regulate several inflammatory processes; such as the production of pro-inflammatory cytokines and immune cell activation and its inhibition will constitute a considerable anti-inflammatory approach.

On the other hand, another molecular mechanism was exerted by *β*-sitosterol and fucosterol by increasing the expression of the Nrf2/HO-1 pathway [25, 64]. Activation of this pathway can be induced upon exposure of cells to inflammatory or OS via decreased production of pro-inflammatory cytokines, as well as production of free radicals and ROS, which may help mitigate cell damage induced by these stresses and protect against multiple diseases associated with inflammatory and/or oxidative stress.

The regulation of the inflammatory response may also be influenced by the presence of HIF-1α, a protein that plays an important role in the cellular response to hypoxia. Studies have shown that this protein is increased in immune cells, such as macrophages when exposed to inflammatory stimuli [109]. Accordingly, suppression of HIF-1α accumulation may have beneficial effects in the regulation of inflammation by reducing inflammatory cytokine synthesis. Fucosterol is a substance that has been shown to inhibit the accumulation of HIF-1α and thereby alleviate inflammation [103].

These findings clearly highlight that the cellular and molecular mechanisms involved in the anti-inflammatory effects of phytosterols are complex and mediate several signaling pathways regulating the inflammatory response. Thus, these plant compounds represent a promising avenue for preventing and managing inflammation and, by extension, CVDs.

### Hypocholesterolemic mechanisms

Exploration of discrepancies in the absorption, metabolism, and pharmacological effects of cholesterol and phytosterols dates back several decades. Early observations revealed that phytosterols were largely excluded from general absorption, but this did not deprive them of their biological effects [115]. Indeed, these compounds have been associated with several biological properties, including their anti-inflammatory and antioxidant capacities. The most common and important property of phytosterols is their ability to decrease blood cholesterol levels, thereby helping to lower the risk of CVDs. The mechanisms that explain this property have been widely studied, focusing on various pathways directly involving cholesterol, such as gene regulation, protein-mediated absorption, interaction with digestive enzymes, and intestinal solubility.

In contrast, atherosclerosis is identified as a major risk factor for CVDs. Thus, the prevention and treatment of this arterial condition are essential to reduce the risks associated with CVDs. Indeed, when atherosclerotic plaques (plaques of cholesterol, fat, etc.) obstruct the coronary arteries, this can lead to myocardial ischemia linked to other cardiac pathologies (heart failure, myocardial infarction, angina pectoris, etc.). Therefore, lowering atherosclerosis risk markers (LDL-cholesterol, HDL-cholesterol, total cholesterol, triglycerides, total cholesterol/HDL-cholesterol ratio, etc.) will be an important preventive approach against CVDs.

For this reason, the hypocholesterolemic potential of several phytosterols from different sources was investigated (Table 3). Indeed, 24-methylenecholesterol, *β*-sitosterol, *β*-sitostanol, stigmasterol, and ergosterol reduced the levels of several markers of atherosclerosis risk [14–17, 30, 94, 116–130]. *β*-sitostanol, in turn, plays a crucial role in preserving cardiovascular health by preventing the formation of atherosclerosis, which results from the accumulation of cholesterol-rich plaques along arterial walls [131]. Likewise, a ratio called "atherogenic index" reflecting the balance between the levels of HDL-C and LDL-C in the blood and often used as a marker of cardiovascular risk was decreased following treatment with *β*-sitosterol [118]. Higher values of this index indicate a higher risk of developing CVDs and atherosclerosis.

**Table 3:** Hypocholesterolemic properties of phytosterols

Molecules	Origins	Experimental methods	Key findings	Refs.
24-methylenecholesterol	*Tapes philippinarum*	Male Wistar rats Cholesterol concentration determination	Decreased the cholesterol level in both serum and liver	[129]
*β*-sitosterol	Purchased	In vivo dual isotope ratio method In situ ligated loop method	Inhibited cholesterol absorption	[126]
–*β*-sitosterol	Purchased	Rats fed butter fat Lipid and apolipoprotein analysis	Lowered the liver cholesterol level	[143]
–*β*-sitosterol	Purchased	Male Wistar rats Micellization study (in vivo and in vitro)	Restricted the micellar solubility of cholesterol Reduced the cholesterol content in the aqueous phase of rat intestinal contents	[136]
–*β*-sitosterol	Purchased	36 male adult F1B hamsters fed for four weeks on a diet rich in saturated fatty acids	Decreased plasma levels of total cholesterol (33%), triglycerides (49%) and the total cholesterol/HDL-cholesterol ratio (48%)	[127]
–*β*-sitosterol	Purchased	L6 cells Determination of lipid contents and fatty acid oxidation rate	Reduced intracellular levels of cholesterol and triglycerides	[121]
–*β*-sitosterol	Purchased	30 or 50 mg Cholesterol-enriched diet [4-^14^C]-cholesterol administered intragastrically once daily for 3 consecutive days	Reduced cholesterol uptake (30%) Lowered total blood cholesterol levels Reduced the atherogenic index	[118]
–*β*-sitosterol	*Citrullus colocynthis*	Dose: 80 mg/10 mL Male domestic rabbits	120 h after the first administration: Reduced serum total cholesterol and triglyceride levels	[144]
–*β*-sitosterol	*Ficus asperifolia*	Cadmium chloride (CdCl_2_)-induced hypertensive rats Spectrophotometric analysis Measurement of atherogenic and coronary heart indices	Reduced markers of risk of atherosclerosis (LDL-cholesterol, triacylglyceride, and total cholesterol)	[125]
–*β*-sitosterol	Purchased	Doses: 100 and 10 μM CCK receptor-bearing cell lines Receptor binding assays	Improved the defective signaling of the CCK1R present in high cholesterol	[137]
–*β*-sitosterol	Purchased	Mice fed a high-fat western-style diet (HFWD) for 17 weeks Lipidomic analysis of liver and serum samples qRT-PCR analysis	Lowered cholesterol, triacylglycerols, and hepatic total lipids levels Increased fecal lipid levels Altered expression of genes involved in lipid metabolism	[14]
–*β*-sitosterol	*Candida rugosa*	Dose: 220-mg/5 mL oil/kg b.w 40 male Golden Syrian hamsters Morphological analysis of epididymal adipose tissues Determination of lipid levels in the serum and liver Analysis of fecal cholesterol	Reduced serum total triglyceride and cholesterol levels Reduced epididymal adipocyte size Protected hepatic polyunsaturated fatty acids Enhanced fecal cholesterol excretion	[30]
–*β*-sitosterol	Purchased	Nanostructured lipid carrier (NLC) formulations hypercholesterolemic mouse model	Reduced the total cholesterol and LDL-cholesterol plasma levels	[17]
–*β*-sitosterol	Purchased	20-mg/kg p.o High-fat diet (HFD)-induced insulin resistance in gastrocnemius muscle Serum lipid profile	Stabilized lipid profile	[57]
*β*-sitostanol	Prepared	Young male Wistar rats Determination of liver cholesterol and triglyceride concentration	Exhibited significantly greater hypocholesterolemic effect than *β*-sitosterol	[128]
–*β*-sitostanol	Purchased	Oral or intravenous administration to rats Lipid analysis	Exhibited a cholesterol-lowering effect superior to that of *β*-sitosterol	[124]
–*β*-sitostanol	Prepared	Rats fed diets high in cholesterol	Exhibited significantly greater hypocholesterolemic effect than *β*-sitosterol	[122]
–*β*-sitostanol	Prepared	Male Japan white rabbits given a cholesterol-supplemented diet	Exhibited significantly greater hypocholesterolemic effect than *β*-sitosterol Decreased LDL-cholesterol Decreased liver cholesterol Prevented more effectively the formation of dietary cholesterol-induced atheroma	[131]
Stigmasterol	Prepared	Day-old white Leghorn cockerels Determination of serum cholesterol levels	Exhibited a barely significant hypocholesterolemic effect	[117]
–Stigmasterol	Not reported	Dose: 50 mg Endogenous cholesterol absorption assessed by including tracer cholesterol in the administered test emulsion (in vivo)	Inhibited cholesterol absorption (54%)	[130]
–Stigmasterol	Purchased	0.5% stigmasterol for 3 weeks 12 wild-type Kyoto (WKY) and 12 Wistar rats	Lowered plasma cholesterol levels (11%) Inhibited intestinal cholesterol Suppressed hepatic cholesterol	[116]
Fucosterol	*Ecklonia stolonifera*	3T3-L1 pre-adipocytes Oil Red O staining	Reduced lipid contents Decreased the expression of the adipocyte marker proteins (C/EBPα and PPARγ)	[132]
–Fucosterol	Purchased	Mouse 3T3-L1 preadipocytes Western blot assay RT-PCR analysis	Inhibited adipogenesis through the activation of AMPK and Wnt/*β*-catenin signaling pathways	[135]
Ergosterol	*Erigeron annuus* L	Dose: 0.23 mM ACAT (acyl-CoA: cholesterol acyltransferase) activity assay Lp-PLA_2_ activity assay	Inhibited ACAT-1 (51.6 ± 0.9%) and ACAT-2 (16.0 ± 0.6%) activity Inhibited Lp-PLA_2_ (51.7 ± 1.2%) activity	[138]
–Ergosterol	*Agaricus bisporus*	In vitro digestion model Caco2 cell cultures Plasma and liver lipid analyses Tissue and feces collection	Exhibited an effective hypocholesterolemic effect	[119]-
–Ergosterol	*Agaricus bisporus*	In vitro digestion model Caco2 cell cultures Plasma and liver lipid analyses Tissue and feces collection	Lowered hepatic triglyceride Modified mRNA expression of cholesterol-related genes	[120]
–Ergosterol	*Agaricus bisporus*	Eighty male albino rats fed HFD Ergosterol (100 mg/kg) Niacin (8.5 mg/kg) 8 week treatment	Lowered the levels of triacylglycerol, total cholesterol, LDL-cholesterol Ergosterol + Niacin were more potent than the agent alone	[16]
–Ergosterol	Purchased	1.5% ergosterol, 8 weeks Sprague–Dawley rats Measurement of serum lipids and associated biochemical parameters Liver lipid measurement Fecal cholesterol and triacylglycerol analysis qRT-PCR analysis	Decreased serum total cholesterol (19.4–21.6%) and LDL-cholesterol (42.0–42.6%) Reduced liver cholesterol (46.8–53.2%) Increased fecal cholesterol excretion (51.0–59.3%)	[94]
–Ergosterol	*Lentinula edodes*	Mice with a hypercholesterolemic diet	Reduced cholesterol levels in the dietary mixed micelles Ergosterol + Supercritical fluid extraction (SFE) extract showed no significant difference in serum cholesterol levels	[15]
–Ergosterol	*Ganoderma lucidum*	3T3-L1 cell cultures and differentiation MTT assay Oil red O staining assay Western blot analysis qRT-PCR analysis	Inhibited lipid droplet synthesis Inhibited C/EBPα and PPARγ expression Inhibited the expression of lipogenic factors: acetyl-CoA carboxylase (ACC), fatty acid translocase (FAT), and fatty acid synthase (FAS)	[134]

Furthermore, inhibiting intestinal cholesterol absorption and suppressing hepatic cholesterol production are two key mechanisms in regulating cholesterol levels in the body. Batta et al. [116] identified stigmasterol as having both of these effects. The principle of dietary cholesterol inhibition in the small intestine is based on the formation of cholesterol-phytosterol complexes that cannot be effectively absorbed. These complexes are then eliminated through the stool, thus reducing cholesterol absorption into the blood. While the principle of suppressing hepatic cholesterol relies on reducing the liver's cholesterol production, which is responsible for both the breakdown of cholesterol from the bloodstream and endogenous cholesterol production; leading to decreased circulating cholesterol levels.

Moreover, ergosterol altered the mRNA expression of cholesterol-related genes according to the study by Gil-Ramírez et al. [120]. In fact, some of these genes are involved in the enzymatic steps of cholesterol synthesis, while others are involved in its transport and metabolism, including absorption, endocytosis, and recycling. By modifying the expression of these genes, we have the ability to directly influence the synthesis, transport, and metabolism pathways of cholesterol in the body.

Additionally, a comprehensive atherosclerosis risk assessment should take into account all risk factors, including lipid markers. In fact, promising results were obtained with *β*-sitosterol, fucosterol, and ergosterol. For *β*-sitosterol, Feng et al. [14]recorded a decrease in hepatic total lipid levels with an increase in fecal lipid levels, whereas Krishnan et al. [57] noted the stabilization of the lipid profile. In addition, fucosterol reduced lipid content [132] and ergosterol inhibited the synthesis of lipid droplets [133]. Similarly, Feng et al. [14] showed that *β*-sitosterol is able to alter the expression of genes related to lipid metabolism, which supports the hypocholesterolemic effect of this substance. Indeed, certain genes are implicated in regulating the degradation/synthesis of lipids, in particular cholesterol. By modulating the expression of these genes, it is possible to reduce blood cholesterol content.

**Fig. 4:**
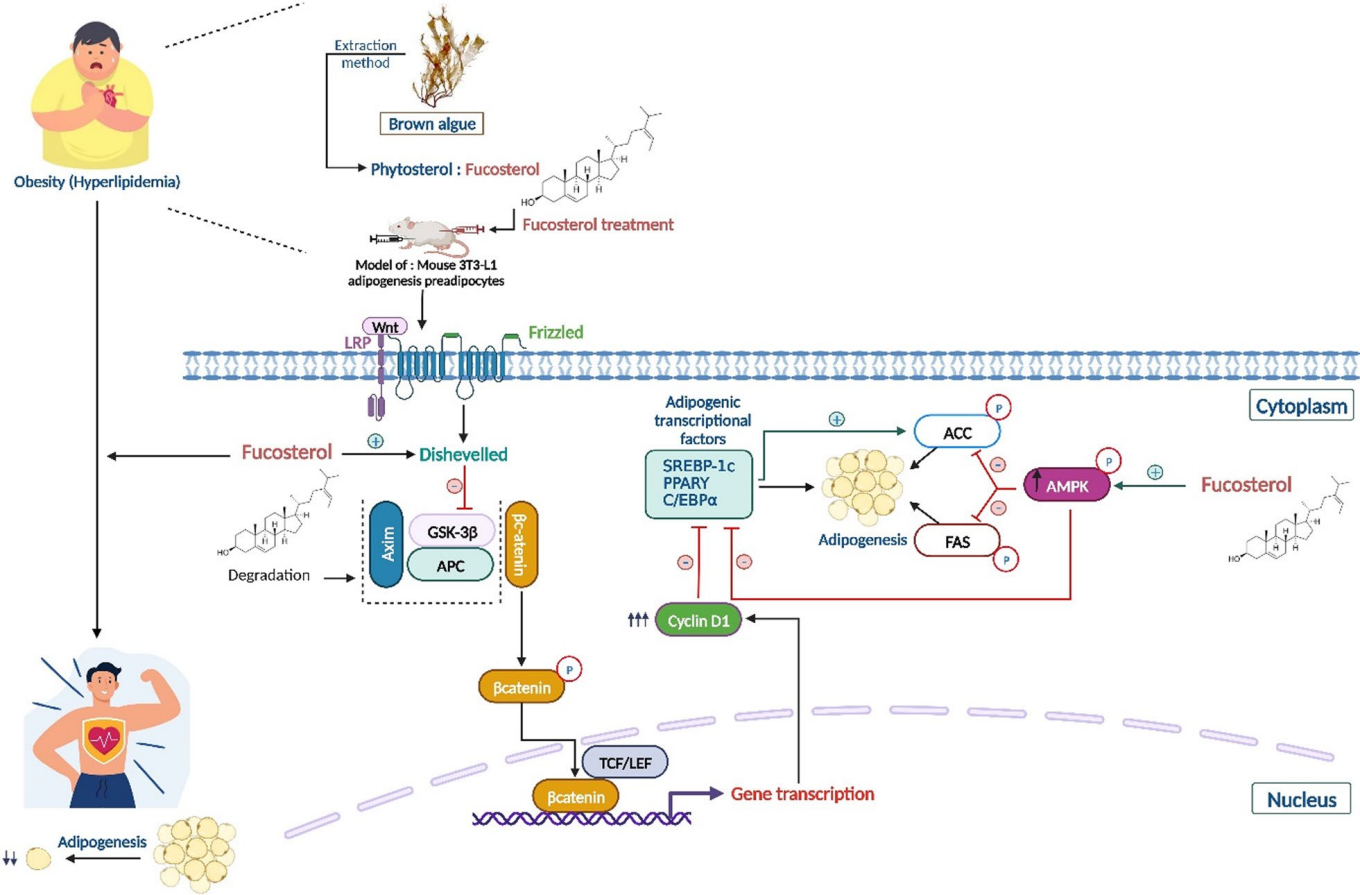
Proposed molecular mechanisms by which fucosterol acts to suppress adipogenesis via activation of the AMPK and Wnt/*β*-catenin pathway. Using mouse 3T3-L1 preadipocytes, fucosterol enhances disheveled expression levels, which in turn inactivates GSK3*β* by increasing disheveled levels. By completely knocking down the GSK-3*β*-Axin-APC complex, the canonical Wnt/*β*-catenin signaling pathway ubiquitinates *β*-catenin and thus inhibits adipogenic development. The connection between Axin and disheveled is triggered by the Wnt molecule, which binds to membrane co-receptors like frizzled and LRP 5/6. *β*-catenin, which is upregulated and can be translocated into the nucleus by DVL2. PPARγ, C/EBPα, and SREBP-1c are adipogenic transcription factors that are inhibited by *β*-catenin, resulting in the upregulation of its target gene, cyclin D1. This is due to the interaction that breaks down the GSK3*β*-Axin-APC complex. The development of preadipocytes into mature adipocytes through adipogenic differentiation is inhibited by the down-regulation of C/EBPα and PPARγ. AMPK was successfully activated by fucosterol, which limits fatty acid synthesis by suppressing ACC and FAS and regulates adipogenesis by blocking the expression of adipocyte master transcription factors (PPARγ, C/EBPα, and SREBP-1c). Therefore, fucosterol can be used as a valuable natural therapeutic ingredient in the management and prophylaxis of CVDs due to its hypocholesterolemic effect. ACC: acetyl-CoA carboxylase; FAS: FA synthase; PPARγ: peroxisome proliferator-activated receptor γ; AMPK: adenosine monophosphate (AMP)-activated protein kinase; GSK3*β*: glycogen synthase kinase 3*β*; C/EBPα: CCAAT/enhancer binding protein α; SREBP-1c: sterol regulatory element binding protein-1c; APC: adenomatous polyposis coli; TCF: T cell factor; LEF: lymphoid enhancer factor; LRP: low-density-lipoprotein-receptor-related-protein

Furthermore, following treatment with ergosterol, the expression of proteins/markers involved in fat accumulation and adipocyte differentiation, namely PPARγ (peroxisome proliferator-activated receptor γ) and C/EBPα, was reduced [134]. This led to a reduction in the accumulation and formation of adipocytes, consequently leading to a reduction in cholesterol levels. Increased expression of these proteins is often linked to increased fat storage, including cholesterol, as well as increased number and size of adipocytes. Likewise, inhibition of the expression of these two proteins was previously recorded with another phytosterol, fucosterol [132]. Moreover, this compound inhibited adipogenesis by activating two essential signaling pathways; Wnt/*β*-catenin and AMP-activated protein kinase (AMPK), both implicated in cell metabolism and various physiological processes, including the regulation of adipogenesis [135]. Indeed, when AMPK is activated, it inhibits lipid synthesis and reduces new adipocyte formation. This leads to the use of lipid stores for energy production (energy metabolism regulation), as well as to the reduction of cholesterol accumulation in adipose tissue (hypocholesterolemia). Similarly, activation of the Wnt/*β*-catenin pathway also reduces the formation of new adipocytes and the accumulation of cholesterol in adipose tissue, but this is by inhibiting the differentiation of mesenchymal stem cells into mature adipocytes (Fig. 4).

Interestingly, recent experiments have highlighted other hypocholesterolemic potentials and mechanisms of *β*-sitosterol, namely reduction in the size of epididymal adipocytes, protection of hepatic polyunsaturated fatty acids (PUFAs), and enhancement of fecal cholesterol excretion [30]. In some cases of lipid imbalance or hypercholesterolemia, it is notable that adipocytes can excessively accumulate lipids, including cholesterol, leading to an increase in their size and, consequently, elevated cholesterol levels. For the mechanism of protecting hepatic PUFAs, it has been shown that by preserving these acids from oxidation or excessive lipid accumulation, healthy liver function and proper regulation of lipid metabolism can be maintained, which can help lower cholesterol. Regarding fecal excretion of cholesterol, it refers to the elimination of cholesterol through the stools in order to reduce its intestinal absorption, thereby helping to maintain balanced cholesterol levels in the body. This property was also observed in the study conducted by He et al. [94] using ergosterol.

Other cholesterol-lowering mechanisms have also been observed with various phytosterols. A study demonstrated that *β*-sitosterol restricts the micellar solubility of cholesterol [136]. It should be noted that the micelles have an important role in the cholesterol absorption process. Indeed, once in the small intestine, dietary cholesterol is emulsified by bile acids, forming micelles, which are small aggregates of cholesterol, and other lipids that facilitate the absorption of lipids by enterocytes. Then the cholesterol contained in the micelles is taken up by the enterocytes and can either return to the intestinal lumen or be incorporated into the chylomicrons for transport. In our context, phytosterols interfere with the stability or formation of cholesterol-containing micelles, thereby decreasing cholesterol uptake. Another cholesterol-lowering mechanism of this phytosterol (*β*-sitosterol) was investigated in a study performed by Desai et al. [137] who demonstrated an improvement in defective CCK1R [cholecystokinin (CCK) type 1 receptor of gastrointestinal tract cells] signaling present in high cholesterol. This receptor is involved in cholesterol absorption and fat digestion, while once activated it causes a series of processes (gallbladder contraction, bile release, and pancreatic secretion stimulation) that favor the absorption of fats, including cholesterol. In certain contexts of hypercholesterolemia, there may be a decrease or dysfunction in CCK1R signaling, leading to an impairment of the aforementioned processes. However, phytosterols intervene by enhancing this defective CCK1R signaling, by optimizing or restoring the function of this receptor, which constitutes the underlying mechanism.

In contrast, inhibition of the activity of certain enzymes such as Lp-PLA_2_, ACAT-1 (acyl-CoA: cholesterol acyltransferase-1), and ACAT-2 by ergosterol was associated with a cholesterol-lowering effect [138]. In their study, Leon et al. [139] classified ACAT inhibitors as anti-atherosclerotic and hypolipidemic drugs. These ACATs are enzymes responsible for converting free cholesterol into cholesterol esters, stored in lipoproteins and tissues, including adipose tissues. Therefore, by inhibiting the activity of ACAT-1 and ACAT-2, the formation of cholesterol esters is reduced; thereby decreasing cholesterol stored in adipose tissues and lipoproteins and subsequently blood cholesterol levels. While Lp-PLA_2_ is an enzyme that plays a role in the modification of lipoproteins, particularly LDL, which promotes their retention in blood vessel walls and thus intervening in atheromatous plaque formation. By inhibiting the activity of this enzyme, it is possible to block these phenomena and, consequently, reduce the level of circulating cholesterol.

Recently, it has been observed that this ergosterol has the ability to inhibit the expression of certain lipogenic factors, in particular enzymes involved in the synthesis of FAs such as acetyl-CoA carboxylase (ACC) and FA synthase (FAS), as well as other enzymes involved in their transport, such as FA translocase (FAT) [134]. Consequently, inhibition of ACC and FAS reduces the biosynthesis of FAs, which are necessary for cholesterol formation, thus contributing to hypocholesterolemia. While inhibiting FAT can reduce the entry of FAs into cells, thereby reducing the availability of substrates necessary for cholesterol formation.

On the other hand, studies have evaluated the combined effect of phytosterols with other nutrients. Interestingly, ergosterol in combination with niacin, also known as vitamin B3, demonstrated a more potent cholesterol-lowering effect than when used individually [16]. This highlights the importance of further preclinical research on the combined use of phytosterols with other therapeutic agents in cholesterol management, which could have implications for the development of therapeutic strategies and nutritional interventions aimed at reducing the risk of cholesterol-related CVDs.

In addition, the hypocholesterolemic potential of phytosterols was clinically investigated and confirmed. As for *β*-sitosterol, it was administered at a dose of 250 mg/kg/day alone or combined with chenic acid (20 mg/kg/day) in seven patients with gallstones in order to assess the level of Bile cholesterol saturation and cholesterol absorption for a period of six weeks. The results showed that *β*-sitosterol alone significantly reduced cholesterol absorption without significant bile desaturation or a synergistic effect with chenic acid [140]. In another study, this phytosterol (10 g/day), supplemented with soy protein, was provided to twenty moderately hypercholesterolemic subjects during 3 separate periods of 40 days each [141]. At the end of these periods, a decrease in triglyceride and LDL-C levels were noted with values of 0.09 ± 0.31 and 0.45 ± 0.30 mmol/L, respectively, associated with a mean increase in plasma HDL-C concentrations (0.12 ± 0.25 mg/dL). These results suggest that adding *β*-sitosterol to low doses of soy protein may be a therapeutic and safe strategy to modestly reduce LDL-C (< 15%) in patients with elevated cholesterol levels.

For the *β*-sitostanol ester, it was supplemented with a basal diet containing soy sterol esters, corresponding to 1.5 g/day of plant sterols, in order to measure hepatic cholesterol synthesis and cholesterol absorption in the small intestine of seven subjects with an ileostomy [142]. Therefore, during the *β*-sitostanol ester period, cholesterol absorption decreased from 56 to 39%, supporting the use of this *β*-sitostanol derivative as a practical option to decrease cholesterol absorption and thus lower its serum levels in the small intestine in people with hypercholesterolemia.

Overall, these results highlight the potential of phytosterols to reduce cholesterol absorption and improve lipid profile. These compounds can be used as dietary supplements or integrated into specific diets to help control cholesterol levels in people suffering from hypercholesterolemia, thus contributing to the prevention of CVDs. However, further research is needed to better understand their mechanisms of action and determine optimal dosages to achieve beneficial effects while ensuring safety.

### Other protective mechanisms

Numerous in-depth investigations have highlighted several other properties of phytosterols that may play a crucial role in the prevention of CVDs. Indeed, *β*-sitosterol was responsible for several effects highly involved in the prevention of these conditions, namely the improvement of immune and endothelial functions and the modulation of certain signaling pathways involved in the regulation of inflammation and lipid metabolism. Murine macrophages were treated for 24 h with *β*-sitosterol (8 μM) combined with vitamin D3 (80 nM) to assess the efficacy of this combination in boosting vitamin D immune function [145]. Vitamin D has been shown to play a role in modulating the macrophage immune system, and its deficiency is a commonly encountered problem [146]. The researchers observed that phytosterol alone reduced cell proliferation (62%), while the vitamin alone was not effective. Importantly, their combination exhibited a 75% reduction in cell proliferation. This suggests that this combination potentiates vitamin D action on macrophage immune function.

Furthermore, using human umbilical vein endothelial cells, Lee et al. [147] associated the effect of the plant *Lespedeza cuneata* renowned for its endothelial dysfunction ameliorating effects as well as various preventive properties with its own two phytosterols, namely *β*-sitosterol 6′-linolenoyl-3-*O*-*β*-_D_-glucopyranoside and *β*-sitosterol. These compounds were responsible for increasing endothelial nitric oxide synthase (eNOS) phosphorylation and NO production in the phosphoinositide 3-kinase/Protein kinase B (PI3K/Akt) signaling pathway, contributing to their beneficial role in preventing CVD-associated endothelial dysfunction. Regarding the modulation of signaling pathways, *β*-sitosterol exhibited cardioprotective effects; by protecting against myocardial ischemia/reperfusion (I/R) damage (in vivo) and cardiomyocyte damage caused by hypoxia/reoxygenation (H/R) (in vitro) [148]. These effects were associated with modulation of PPARγ/NF-κB signaling during myocardial I/R injury. Recently, El-Shoura et al. [149] investigated the effectiveness of a combination therapy of *β*-sitosterol with trimetazidine (TMZ) to attenuate experimentally induced cardiotoxicity (in vivo). Study results showed that the administration of *β*-sitosterol alone or combined with TMZ provides high protection against cardiotoxicity by decreasing oxidative stress as well as inflammatory and apoptotic biomarkers.

On the other hand, some CVDs, such as strokes and heart attacks, are often caused by the formation of blood clots that clog vessels. This clot formation results from clumping of blood platelets; cells responsible for platelet aggregation. Therefore, the inhibition or reduction of this platelet aggregation essentially contributes to the prevention of CVDs. In this sense, using a model of platelet aggregation induced by different inducers, Feng et al. [69] proved in vitro that ergosterol exhibits a promising anti-platelet aggregation effect, in a concentration-dependent manner. Another phytosterol, called brassicasterol, exerted a cardiovascular protective effect by significantly inhibiting human angiotensin-converting enzyme (12.3 μg/mL; 91.2% inhibition), which plays a key role in blood pressure regulation [150].

## Concluding remarks and perspectives

Since CVDs are a major contributor to global mortality, their management represents a public health priority. This approach requires individual management of the different risk factors, with particular importance given to nutrition before treatment. Based on the findings of several studies assessing the antioxidant, anti-inflammatory, and cholesterol-lowering potential of various phytosterols, highlighting the underlying mechanisms of action. Indeed, most of these investigations have demonstrated that these natural components exert their anti-inflammatory, antioxidant, and hypolipemic effects as well as other properties at different levels via cellular, sub-cellular, and molecular mechanisms.

These outcomes also highlight the possibility of incorporating phytosterols in the formulation of new drugs for the prevention and management of CVDs. It is essential to note, however, that these trials were conducted in a preclinical setting, and that further clinical research is needed to confirm these conclusions and their practical applications.

Although phytosterols have a relatively well-established but moderately reproducible dose-dependent hypercholesterolemic action, their position in the therapeutic arsenal remains uncertain, mainly due to a lack of recognition. Given the lack of evidence regarding their benefit/risk ratio in the general population, it does not seem appropriate to extend their application to individuals with normocholesterolemia. This is why, for the first time, the updated guidelines of the European Society of Cardiology/European Atherosclerosis Society (ESC/EAS) for the treatment of disorders of lipid metabolism propose the use of plant sterols as part of lifestyle adjustments aimed at lowering blood cholesterol concentrations. Recently published genetic research reveals that plant sterols are inherently atherogenic. As the German Society of Cardiology (DGK) recently stated, randomized controlled trials with concrete cardiovascular outcomes are needed before advocating the use of plant sterols to lower serum cholesterol.

## Data Availability

Data sharing is not applicable to this article as no new data were created or analyzed in this study.
